# Discovery of novel α-amylase inhibitors using structure-based drug design

**DOI:** 10.1186/1758-2946-6-S1-P50

**Published:** 2014-03-11

**Authors:** Jamil Al-Asri, Gerhard Wolber

**Affiliations:** 1 Computer-Aided Molecular Design, Pharmaceutical Chemistry Department, Freie Universität Berlin, Berlin, 14195, Germany

## 

α-Amylase is an endoamylase and belongs to glycoside hydrolase family 13 (GH 13) according to the classification of carbohydrate-active enzymes [1]. It initiates starch hydrolysis into smaller oligomers. Inhibitors of this enzyme are of pharmacological importance as α-amylase is considered as attractive target for treating elevated post-prandial blood glucose levels resulting in obesity and type II diabetes. Besides the application as a drug, it is highly interesting to classify nutritional components, such as food additives or secondary plant metabolites with respect to their modulation of α-amylase.

**Figure 1:**
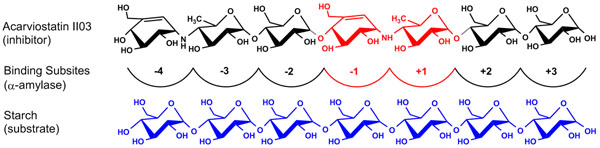
Subsites of α-amylase with Acarviostatin II03 inhibitor, (ki ~ 14 nM) (PDB entry: 3OLE) and starch. Site of cleavage is between subsites -1 and +1.

We present a model that predicts the affinity of small organic molecules to α- amylase. On the basis of available crystal structures (Figure 1) [2], we developed a virtual screening workflow for the identification of novel non- peptidic, non-carbohydrate α-amylase inhibitors. In addition to virtual screening using structure-based 3D pharmacophore models [3], molecular docking and clustering for diversity selection have been applied as post-screening filters. Fourteen virtual hits were purchased and tested in vitro using a kinetic assay with p-Nitrophenyl-α-d-maltopentaoside (PNPG5) as a chromogenic substrate. Three of those fourteen compounds showed concentration-dependent inhibition with promising IC_50_ values (hit rate: 21%).
